# VOC Removal Performance of a Joint Process Coupling Biofiltration and Membrane-Filtration Treating Food Industry Waste Gas

**DOI:** 10.3390/ijerph16173009

**Published:** 2019-08-21

**Authors:** Krystyna Lelicińska-Serafin, Anna Rolewicz-Kalińska, Piotr Manczarski

**Affiliations:** Warsaw University of Technology Faculty of Building Services, Hydro and Environmental Engineering, 00-653 Warsaw, Poland

**Keywords:** biofiltration, membrane, filter bed, volatile organic compounds (VOCs), efficiency, food industry

## Abstract

This study aimed to assess the efficiency of removal of volatile organic compounds (VOCs) from process gases from a food industry plant in East Poland, producing high-quality animal (goose, duck, and pig) and vegetable fats, using a two-stage method which is a combination of biological purification and membrane-separation. The research, conducted on the semi-technical scale, compared the effects of traditional and two-stage biofiltration carried out under the same process conditions. The concentrations of VOCs in process gases were measured by means of a multi-gas detector. Additionally the temperature and humidity of gases were determined by a thermoanemometer under filter bed, following the EU and Polish National Standard Methods Two different types of filling materials (the mix of stumpwood chips and bark, and the mix of stumpwood chips, bark, and compost) and two types of membranes (three-layer semi-permeable membrane fabrics were used, with differences in air permeability and water tightness) were analyzed. During all processes basic operational parameters, the biofilters were controlled, including surface load, volumetric load, duration of gas contact with the filling layer, flow rate, and pressure drops (in the biofilter and on the membrane). The analyzed gases were characterized by very high variability of VOC concentrations (ranging from 350 ppb to 11,170 ppb). The effectiveness of VOC removal (RE_voc_) was calculated by comparing the analytical results of raw and purified gases. The effectiveness of VOC removal with the application of traditional biofiltration during the experiment varied between 82% to 97% and was related to different parameters of the filling materials (mainly specific surface and moisture), reaching lower value for the mix of stumpwood chips and bark filling. The obtained results showed that the application of membrane improved the efficiency of biofiltration in all the analysed cases from 7% to 9%. The highest effectiveness was obtained using the filter bed in the form of stumpwood chips, bark, and compost in connection with the more permeable membrane. It was maintained between 96% to 99%, reaching an average value of 98%. The selection of the membrane should be determined by its permeability and the values of flow resistance.

## 1. Introduction

Odor nuisance and the emission of contaminants to the atmosphere from technological processes are issues related to the protection of the environment and human life [1,2]. The emission of volatile organic compounds (VOCs) is one of the main problems related to air pollution in selected branches of the industry, among others, in the chemical, textile, metallurgic, and food industries [3,4,5]. In some regions, food processing and animal breeding have been identified as the primary sources of VOC emission [6,7]. Modern research has also addressed the problem of VOC emission related to the preparation of food, particularly meat, in the processes of boiling and frying in large industrial plants [8,9]. According to literature reports, the broadly defined food sector, covering both the production and processing of food, particularly that related to the processing of animal products, constitutes an important source of VOC emission. Therefore, it requires the application of relevant technological and legislative solutions for protection of the quality of the atmosphere by limiting VOC emission from technological processes, including “end-of-pipe” solutions [4,10,11].

For many years, limiting VOC emission in industrial installations has been conducted with the application of biological processes, incorporating bioreactors. Analyses of the operating costs show that biological methods are still a very competitive alternative to physical and chemical methods [12]. Biological purification of gases covers the parallel performance of two stages of purification: Sorption of contaminants and their biological decomposition. Contact of gas with the layer of filter bed covered with moist biofilm leads to sorption of contaminants with their further decomposition by microorganisms [13]. The most frequently applied solutions of biological purification include classic biofilters (horizontal and vertical), bioscrubbers, biological filter beds, and membrane filters [13,14]. The range of technologies for biological purification of industrial gases is continuously expanding. In addition to the parameters directly related to purification efficiency, the economic and operation conditions related to efficient full industrial scale applications are of high importance. Among others, such factors include energy consumption, quantity of post-process waste products, and resistance to atmospheric conditions [15,16]. The most commonly applied solutions for biological purification of gases still include traditional horizontal biofilters which, in accordance with the guidelines of the European Commission (e.g., in the scope of limiting the odor nuisance and emission of VOCs), are included in the solutions of the Best Available Techniques (BAT) [17,18,19,20]. The literature review shows that although the process of biological gas purification by biofiltration may achieve high removal levels of contaminants, including VOCs, research on the improvement of the efficiency of the method is still required. This is due to the fact that process efficiency varies between 60 to 95% in comparison to thermal oxidation (efficiency 95–99%), catalytic oxidation (efficiency 90–98%), absorption (efficiency 90–98%), or membrane separation (efficiency 90–99%) [21]. Nevertheless, traditional biofiltration has many advantages, including low investment and operational costs and no cumbersome waste, and is successfully applied on the technical scale [22,23]. These benefits justify investigation of improvement of the latter methods. The literature review indicates that in the treatment of waste and odorant gases since the end of 2010, there has been an increasing trend in developing bioreactor configurations (including those combined with physical methods) [23,24] to improve the efficiency of removing pollutants from process gases (also VOCs), including those from the food industry [25,26,27]. The literature also clearly indicates that research conducted on the laboratory scale does not take into account all the operational aspects that appear on the industrial scale. Consequently, research on the removal effectiveness of VOCs and abatement of other nuisances in the biofiltration process requires studies on the semi-technical and technical scales. Results obtained in the laboratory, due to the fully controlled conditions affecting the purification process, show higher biofiltration effectiveness than those from research conducted in field conditions. This results from many external and operational factors that may affect the operation of these devices on the full technical scale, such as the influence of atmospheric conditions (temperature, humidity) and temporally variable technological-operational parameters (gas flow rate, contact time with the filling media, biofilter load, dynamic changes in concentration of contaminants in process gases, etc.) [23,28,29,30].

The research described in this paper involves the analysis and assessment of the modified biofiltration process effectiveness in removal of VOCs from the food industry processes. The research employed a pilot two-stage biofilter combining traditional biofiltration and purification on a membrane filter. During the literature review, no research was found which included a combination of traditional biofiltration and purification on a membrane filter. An additional factor investigated during the research was application of two different filling materials, as well as two types of membranes constituting the second stage of gas purification. The subject of the research was twofold: The verification of the effectiveness of two-stage VOC removal from food industry process gases on the semi-technical scale, and the dependencies between the technical parameters and the efficiency of the employed biofilter. Therefore, examination of the technological parameters of the applied filling media was carried out, along with strict control of the operational parameters of the analyzed biofilters (compared to the reference values reported in the literature). The research described in this paper combines two gas purification methods, resulting in the possibility of increasing the effectiveness of VOC removal, and at the same time improving the first stage of purification through the application of the second stage. Moreover, conducting research on the semi-technical scale increases the credibility of the obtained results and constitutes an important contribution in the search for more efficient methods of purification of food industry gases.

## 2. Materials and Methods

### 2.1. Semi-Technical Scale Biofilter

The object of the study is a pilot biofilter allowing use of two purification methods: Traditional (single-stage) biofiltration with the application of an open biofilter, and integrated (two-stage) biofiltration with the second stage of purification in the form of a membrane fabric covering the surface of the device. The examined pilot biofilter was applied on the semi-technical scale, being connected to an installation for the extraction of process gases from a food industry plant located in Eastern Poland which produces high-quality animal (goose, duck, and pig) and vegetable fats.

Animal fat processing involves a series of purifying steps followed by modification into more usable products. The major steps are: Settling and degumming (removal of animal or plant proteins, carbohydrate residues, phosphatides, and water), neutralization/refining with alkali (of nonglyceride fatty materials by washing the oils with strong alkaline water solutions), bleaching, deodorization, fractionation (partial crystallization of a fat or oil at a specific temperature), and hydrogenation (direct addition of hydrogen to double bonds of fatty acids, to modify vast quantities of fats and oils).

**Figure 1:**
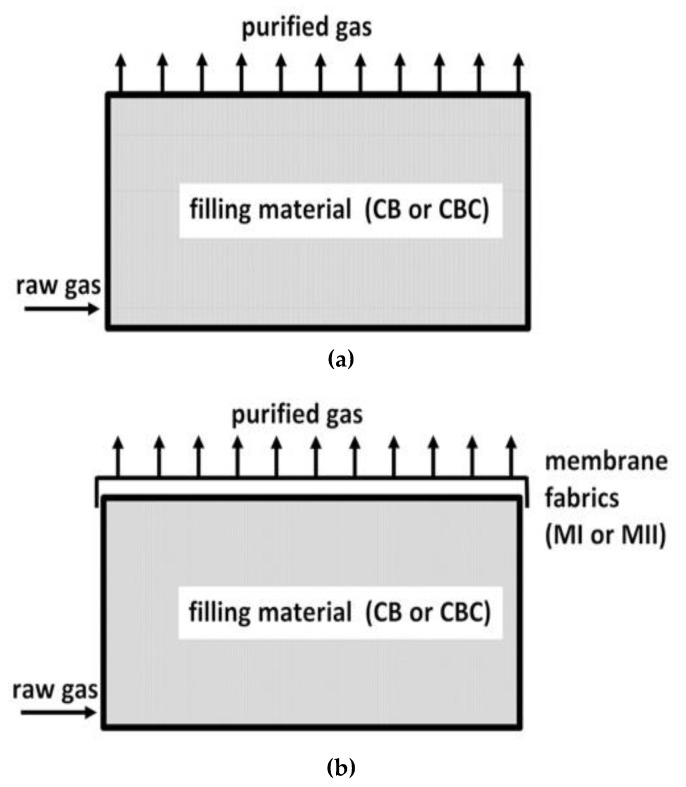
Single-stage (**a**) and two-stage biofiltration (**b**)**.**

The analyzed biofilter (Figure 1) is equipped with a fan, scrubber, automatic regulation of gas flow, instrumentation for measurement of gas flow, temperature, and humidity with data logger downstream the biofilter, systems for distribution of process gases and leachate drainage, membrane mounting system (including sealing), and a sliding shelf, enabling sample collection. The footprint of the active part of the studied biofilter has dimensions of 1.32 m × 3.00 m.

### 2.2. Experimental Design

#### 2.2.1. Experimental Design

The experiment was implemented in six phases, presented in Table 1. The studied device was filled with two types of materials: A mix of stumpwood chips and bark (CB), and a mix of stumpwood chips, bark, and compost (CBC). The thickness of the filter bed 1.1÷1.2 m. The detailed characteristics of the filling materials are described in Section 2.4. For both filling variants, research on single-stage (traditional) and two-stage biofiltration was conducted. Two membrane fabrics (MI and MII) were subsequently used for the second stage of gas purification and their parameters are presented in Section 2.5. The experimental first phase covered research on the effectiveness of traditional biofiltration with the application of CB filling, and then research on the effectiveness of two-stage biofiltration after subsequent covering of the filling with membranes MI and MII. The analogous procedure was performed for CBC filling. The conditioning duration of the filling material was approximately three weeks each time.

#### 2.2.2. Measurement Points and Number of Measurement Series, Samples, and Measurements

**Figure 2:**
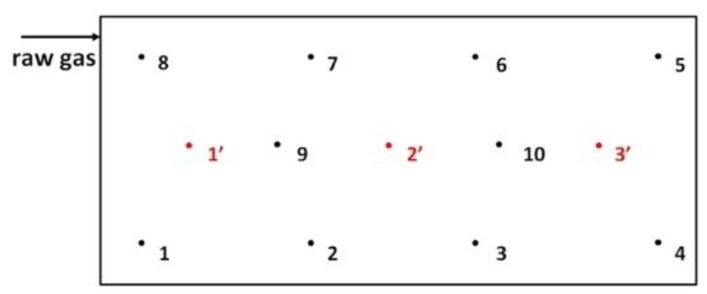
Sample points locations, points 1–10 gases sampling, points 1′–3′ filling material sampling.

During the period May 1, 2018 to July 24, 2019, 20 measurement series were carried out. The number of measurement series resulted from the fact that the experiment was conducted on a semi-technical scale under the authentic operating conditions of the industrial plant. During each measurement series, samples of raw and purified process gases were collected. Raw gases were collected at the inlet to the biofilter (three repetitions in each measurement series) using an intake connector. Purified gases were sampled from 10 points on the surface of the biofilter (three repetitions from each measurement point in each measurement series). Figure 2 shows the locations of the sampling points. Samples of purified gases (from the surface of the biofilter) were collected with the application of a shield eliminating the effect of external conditions, i.e., a glass funnel positioned on the surface of the filling layer inside a metal or plastic cover. The gas sampling pipe was connected to the screened glass funnel. The described gas sampling method ensured isolation and representativeness of the samples.

The effectiveness of VOC removal was calculated by comparing the analytical results of raw and purified gases (11). The results were expressed as removal efficiency of VOCs (RE_voc_). RE_voc_ was calculated as follows:

RE_voc_ (%) = (C_input_ - C_utput_)/C_input_ × 100,

where C_input_ corresponds to the ppb concentration of VOCs in the raw gases, and C_output_ corresponds to the ppb concentration of the purified gases.

Moreover, during the single-stage biofiltration measurement series (series 1–4 and 11–14), samples of the biofilter material filling were collected. During each of the measurement series, samples of the filling were collected from three points in the biofilter (Figure 2). At each sampling point, the filling material was collected from the entire vertical cross-section of the filter bed and averaged. From each averaged sample, a sample for laboratory analysis was prepared. Examination of physiochemical indicators (pH, moisture, specific surface, hydraulic diameter) was repeated three times.

### 2.3. Analytical Methods

The concentrations of VOCs in raw and purified process gases were measured by multi-gas detector MultiRAE (Rae Systems) with sensors based on various measurement principles (electrochemical and catalytic, infrared, photoionization sensor (PID)) with 10 ppb limit of detection and range from 0 to 20,000 ppb.

The temperature and humidity of gases were measured by means of a thermoanemometer TA440 (Airflow Instruments) with limits of detection for temperature (0.1 °C) and humidity (0.1%).

Total moisture, content of organic substances, pH, and grain size composition (by means of sieves with openings: 0.071; 0.1; 0.25; 0.5; 1.0; 2.0; 10.0; 25.0 mm) of the filter bed were determined following the relevant European Union and Polish National Standard Methods [31,32,33,34,35].

Measurements of the specific surface of the filling material were performed in accordance with the method of nitrogen sorption at a liquid nitrogen temperature using a Micromeritics ASAP2020 instrument with detection limits 0.01 m^2^·g^−1^.

The gas flow rate through the biofilter was recorded by the integrated control and measurement instrumentation. The surface and volumetric load of the biofilter, as well as contact time of gases with the filter medium, were calculated using the gas flow rate, active surface of the biofilter, and filter bed thickness.

The flow resistance in the experiment was determined as the difference of pressure before and after the biofilter (or membrane) using a differential pressure gauge.

### 2.4. Filling Material (Filter Bed)

**Figure 3:**
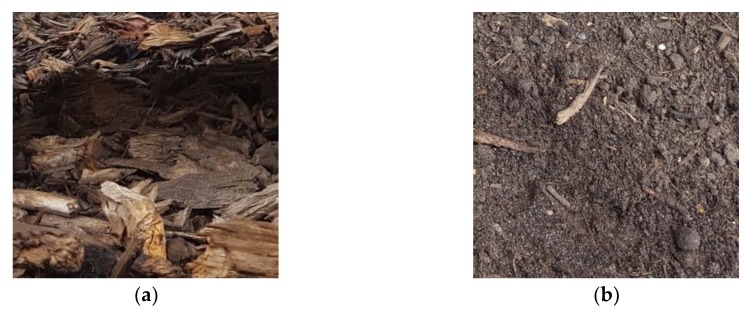
Applied filling materials, (**a**) A mix of stumpwood chips and pine bark (CB), (**b**) a mix of stumpwood chips, pine bark, and green waste compost (CBC).

Two types of materials were used for the filling of the analyzed biofilter: A mix of stumpwood chips with a grain diameter of 20–80 mm (50%) and pine bark (50%)–denoted as CB–and a mix of stumpwood chips with pine bark (50% in total, in a 1:1 ratio) with green waste compost (50%)–denoted as CBC (Figure 3). The thickness of the filter bed was 1.1–1.2 m.

### 2.5. Membrane Fabrics–Second Stage of Purification

The second stage of purification in the studied integrated biofilter employed three-layer semi-permeable membrane fabrics. Their external and internal layers were made of polystyrene, and the middle functional layer was an ePTFE membrane. The membrane, denoted as MI, is characterized by an average air permeability of 17.8 mm·s^−1^ and average watertightness of 199 cm H_2_O. The membrane, denoted as MII, is characterized by an average air permeability of 3.90 mm·s^−1^ and average watertightness of >2000 cm H_2_O.

The required surface of the applied membrane fabrics was calculated based of their permeabilities and surface loads of the biofilter in order to enable unimpeded flow of processed gases in the experimental conditions.

## 3. Results and Discussion

### 3.1. Raw Gases Characteristics

The analyzed gases were characterized by very high variability of concentrations of leading contaminants—VOCs (range from 350 ppb to 11,170 ppb), resulting from the conditions in animal and vegetable fats processing technology. The fluctuations were recorded both in each measurement series and during the whole research cycle. The concentration of organic pollutants in the raw gases did not exceed 1%.

**Figure 4:**
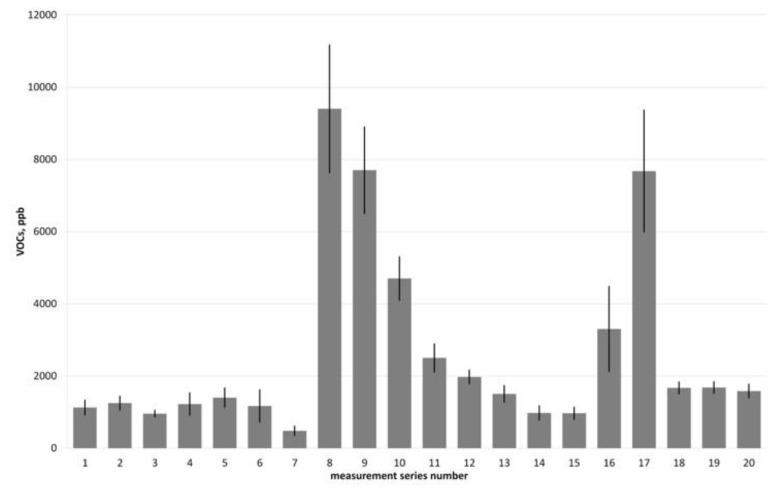
Fluctuations of volatile organic compound (VOC) concentrations in the analyzed raw food industry gases throughout the research cycle. Results for each measurement series are represented by the arithmetic average.

Despite the differentiation mentioned above (especially observed during measurement series no. 8–10 and 17), the proposed configurations of the experiment can be compared because the raw gases came from the same source at all times during the experiment. Their characteristics indicated the possibility of biological purification, and all parameters of the tested biofilter were in the ranges indicated as optimal for the purification process. The differentiation resulted mainly from the technical scale of the experiment and the real industrial conditions (properties of the raw gases). The remaining gas parameters, such as temperature and humidity, were practically unchanged during the course of the study. Figure 4 presents fluctuations of VOC concentrations throughout the research cycle.

The correctness of the basic parameters of the analyzed process gases was confirmed by the possibility of their purification with the application of biological methods. The humidity of the purified gases was maintained close to saturation level and averaged 98.1% (SD ± 3.7%). The range of readings varied from 88.0 to 99.9%. The gas temperature did not exceed 33.2 °C, averaging 22.5 °C (SD ± 6.1 °C). The range of readings was 10.1 ÷ 33.2 °C. In accordance with the operational requirements for biofilters, the inlet gases were humidified to reach saturation state and their temperature remained in the range 0 °C–40 °C [10,16]. The VOC concentrations in raw gases did not exceed levels specified in the literature as limiting the effectiveness of the process (>5000 ppm) [23].

### 3.2. Biofiltration Process Parameters

The majority of the operational parameters of the examined pilot biofilter were within the ranges reported in the literature as correct (Table 2).

During all phases of the experiment, control of the flow rate and pressure drops in the biofilter were also carried out. The results are presented in Table 3. The use of membranes as the next stage of purification did not significantly affect the value of flow resistance. The MI membrane caused a pressure drop in the range from 59 to 64 Pa. For the MII membrane, these values were in the range from 28 to 63 Pa.

### 3.3. Effectiveness of VOC Removal–Influence of the Filterbed (Single-Stage Biofiltration)

**Figure 5:**
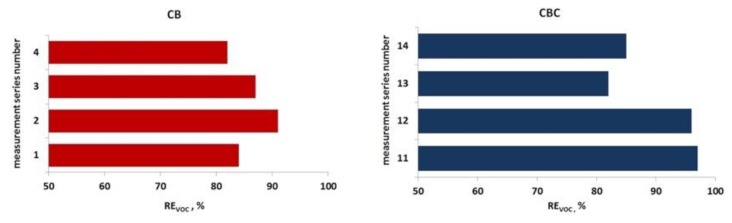
Mean effectiveness of VOC removal in subsequent measurement series of single-stage biofiltration for both fillings.

The efficiency of VOC removal with the application of traditional (single-stage) biofiltration during the experiment varied between 82% to 97% on average, reaching the lower value for the CB filling (86%) and higher value for the CBC filling (90%). Table 4 shows VOC concentrations in the raw and purified gases during subsequent measurement series of single-stage biofiltration. Figure 5 presents the variability of the contaminant removal effectiveness with the application of both filling materials.

**Figure 6:**
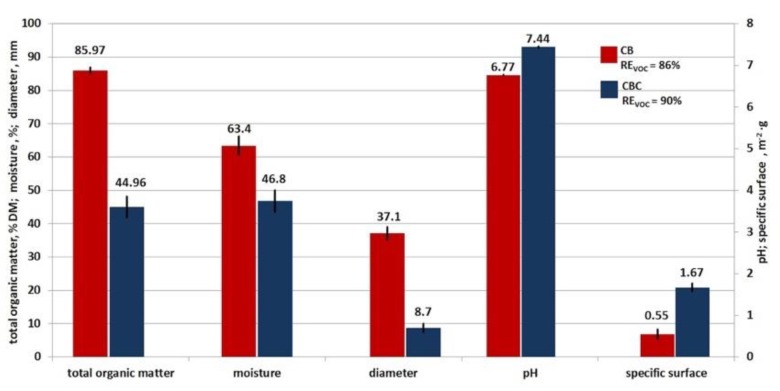
Technical parameters of the used fillings collated with the VOC removal effectiveness–mean values.

The variability of VOC removal effectiveness during single-stage biofiltration is related to different parameters of the applied filling materials. In spite of differences, the parameters are within the optimum ranges reported in the literature for the biofiltration process [22,38]. Figure 6 depicts mean values of the characteristic parameters for both fillings used in the study and mean VOC removal effectiveness.

The effectiveness of VOC removal was affected to the greatest extent by differences in the values of specific surface (and the related hydraulic diameter) and moisture content of both fillings. The mean value of specific surface of the CB filling was at a level of 0.55 m^2^·g^−1^ and was three times lower than for the CBC filling (1.67 m^2^·g^−1^). The mean value of the hydraulic diameter of the CB material was 37.1 mm and was approximately five times higher than the hydraulic diameter of the CBC filling (8.7 mm). Higher efficiency of biofiltration was observed at a larger specific surface (accompanied by a smaller hydraulic diameter). The analysis of mean values of the moisture content of the filling material showed higher effectiveness of the process at lower moisture content and still remained within the optimum range for biofiltration. According to the literature, the moisture content should be below 90% [21], as moisture intake clogs the pores. Table 5 presents basic technical parameters of the filling materials used in the research, compared with optimal values from the literature.

### 3.4. Effectiveness of VOC Removal–Influence of Membranes (Two-Stage/Integrated Biofiltration)

**Figure 7:**
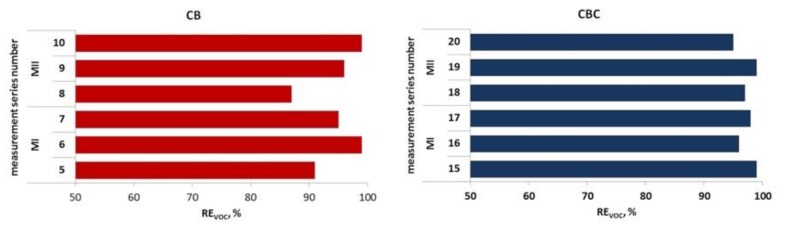
Mean effectiveness of VOC removal in subsequent measurement series of two-stage biofiltration.

The effectiveness of VOC removal by the two-stage biofiltration varied from 88% to >99%, depending on the type of the filling materials and membrane fabrics. Table 6 shows VOC concentrations in raw and purified gases during subsequent measurement series of two-stage biofiltration. Figure 7 presents the variability of the contaminant removal in relation to the phase of integrated biofiltration (for both types of fillings and membranes).

## 4. Conclusions

The experimental results presented here provide a new and important contribution in the search for more efficient methods for biological purification of gases. Such exploration is particularly needed, as the variable concentrations in raw gases create difficulties in both the purification and control of the process. Specifically, the following conclusions can be drawn:

- The application of membrane fabrics as the second stage of purification improved the effectiveness of biofiltration in all the analyzed cases from 7% to 9%.
- In the case of combining both types of membranes (MI and MII) with CBC filling, the effectiveness of VOC removal was more stable than in the case of integrated biofiltration with the application of CB filling.
- The most effective solution for the VOC removal from process gases proved to be an integrated filter with a filter bed in the form of stumpwood chips with bark and compost (CBC), supplemented with the more permeable membrane (MI). The biofiltration effectiveness was maintained between 96% to 99%, reaching an average value of 98%.
- The analyzed pilot integrated biofilter is an innovative solution in which the membrane constituting the cover of the biofilter bed is not only the second stage of purification by itself, but also contributes to the improvement of the first stage processes. Use of the membrane covering the biofilter allows better control of the processes, among others facilitating the maintenance of correct (not too high) humidity content in the biofilter layer. This is extremely important for the process effectiveness–the conducted research showed that the moisture level has a significant effect on the biofiltration efficiency. The effectiveness of VOC removal was higher at lower moisture values and remained within the optimal range.
- The effectiveness of VOC removal varied depending on the applied filling materials. In the case of single-stage biofiltration, the mix of stumpwood chips, bark, and compost proved more beneficial.
- The selection of the filling material in both cases of single-stage and integrated biofiltration should be governed by the parameters important for the biofiltration process–with particular consideration of the specific surface, which plays a substantial role in the sorption process.
- The selection of the membrane fabric, constituting the second stage of purification, should be determined by its permeability (allowing purification of the specified stream of process gases) and the flow resistance values, to eliminate the risk of gas leaks without purification.
- The research was conducted on the semi-technical scale, so it constitutes an important source of credible results.
- Further research on the membranes studied in this paper should be carried out to establish their effect on the flow resistance values and on the filling material changes, resulting from covering with membrane fabrics.
- Further research is carried out in the optimal variant (CBC + MI) identified in the article on the integrated biofilter in full technical scale.

## Figures and Tables

**Figure 8:**
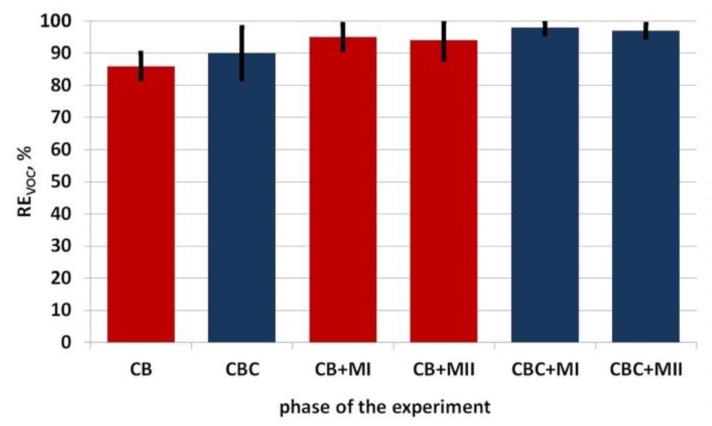
Changes in the effectiveness of VOC removal with the application of single-stage and integrated biofiltration depending on the type of biofilter bed and membrane.

**Table 1:** Comparison of measurement series in particular phases of the experiment.

Filterbed	Membrane Type	Numbers of Measurement Series	Identification of Experiment Phase
mix of stumpwood chips and bark (CB)	-	1–4	CB
	MI	5–7	CB + MI
	MII	8–10	CB + MII
mix of stumpwood chips, bark and compost (CBC)	-	11–14	CBC
	MI	15–17	CBC + MI
	MII	18–20	CBC + MII

**Table 2:** Comparison of the basic operational parameters of the studied pilot biofilter.

Operational Parameters		CB *	CBC *	CB + MI *	CBC + MI *	CB + MII *	CBC + MII *	Recommended Values
Surface load m^3^·m^−2^·h^−1^	average value ± SD	96.5 ± 6.1	81.3 ± 21.7	97.1 ± 4.2	99.4 ± 1.0	97.8 ± 1.0	100.7 ± 1.1	45 ÷ 150 [36,37]
	range	87.4 ÷ 100.5	62.4 ÷ 100.5	92.4 ÷ 100.3	98.7 ÷ 100.5	97.0 ÷ 99.0	99.5 ÷ 101.5	
Volumetric load m^3^ m^−3^·h^−1^	average value ± SD	84.0 ± 5.3	70.7 ± 18.9	84.5 ± 3.6	86.5 ± 0.8	85.1 ± 0.9	87.6 ± 0.9	5 ÷ 500 [38]
	range	76.0 ÷ 87.4	54.2 ÷ 87.4	80.4 ÷ 87.2	85.9 ÷ 87.4	84.3 ÷ 86.1	86.5 ÷ 88.3	
Duration of gas contact with the filling layer (s)	average value ± SD	43 ± 3	54 ± 14	43 ± 2	42 ± 1	42 ± 1	42 ± 1	30 ÷ 60 [1,38]
	range	41 ÷ 47	41 ÷ 66	41 ÷ 45	41 ÷ 42	41 ÷ 43	41 ÷ 43	
Filter bed thickness (m)	range	1.1 ÷ 1.2	1.1 ÷ 1.2	1.1 ÷ 1.2	1.1 ÷ 1.2	1.1 ÷ 1.2	1.1 ÷ 1.2	1 ÷ 1.5 [1,38,39]

* Values of parameters for each experimental phase are the arithmetic average of four measurement series (for CB and CBC) and three measurement series (for other phases of experiment). In all measurement series three parallel measurements were conducted.

**Table 3:** Hydraulic parameters of the studied pilot biofilter.

Hydraulic Parameters of the Studied Pilot Biofilter		CB *	CBC *	CB + MI *	CBC + MI *	CB + MII *	CBC + MII *
flow rate m^3^·h^−1^	average value ± SD	382 ± 24	322 ± 86	385 ± 16	394 ± 4	387 ± 4	399 ± 4
pressure drops in biofilter (Pa)	average value ± SD	379 ± 13	596 ± 84	536 ± 129	683 ± 54	469 ± 12	617 ± 48
pressure drops on the membrane (Pa)	average value ± SD	-	-	64 ± 25	59 ± 2	28 ± 25	63 ± 6

* There were four measurement series for CB and CBC and three measurement series for other phases of the experiment.

**Table 4:** VOC concentrations in raw and purified gases during single-stage purification with the application of two types of fillings–for each measurement series.

Experiment Phase	Number of Measurement Series	VOC in Raw Gases (ppb)		VOC in Purified Gases (ppb)	
		Average Value ± SD	Range	Average Value ± SD	Range
CB	1	1130 ± 207	940 ÷ 1350	180 ± 9	170 ÷ 200
	2	1250 ± 190	1060 ÷ 1440	118 ± 9	110 ÷ 130
	3	950 ± 90	860 ÷ 1040	122 ± 8	110 ÷ 130
	4	1220 ± 310	910 ÷ 1530	220 ± 11	210 ÷ 230
	Mean	1138 ± 135	950 ÷ 1250	160 ± 49	118 ÷ 220
CBC	11	2500± 390	2110 ÷ 2890	66 ± 8	50 ÷ 80
	12	1970 ± 190	1780 ÷ 2160	87 ± 7	80 ÷ 100
	13	1500 ± 230	1270 ÷ 1730	269 ± 7	260 ÷ 280
	14	980 ± 200	780 ÷ 1180	146 ± 10	130 ÷ 160
	Mean	1738 ± 650	980 ÷ 2500	142 ± 91	66 ÷ 269

**Table 5:** Technical parameters of the biofilter filling materials–values for each measurement series and mean values.

Filter Bed		CB *	CBC *	Recommended Values
Moisture (%)	min	60,6	42.7	30–60 [14,37,38]
	max	66,3	50.5	
	mean ± SD	63.4 ± 2.9	46.8 ± 3.4	
pH	min	6.75	7.27	6–9 [13,37,40]
	max	6.79	7.7	
	mean ± SD	6.77 ± 0.02	7.44 ± 0.03	
Total organic matter (% d.m.)	min	84.97	40.61	>40 [25,41]
	max	87.53	47.79	
	mean ± SD	85.97 ± 1.10	44.96 ± 3.30	
Specific surface m^2^·g^−1^	min	0.37	1.53	1–100 ** [39,40]
	max	0.67	1.8	
	mean ± SD	0.55 ± 0.13	1.67 ± 0.11	
Hydraulic diameter (mm)	min	34.8	6.7	>4 [25,41]
	max	39.6	9.9	
	mean ± SD	37.1 ± 2.0	8.7 ± 1.4	

* Values of parameters for each measurement series are the arithmetic average of three parallel measurements. ** Range for different filling materials used in biofiltration.

**Table 6:** VOC concentrations in raw and purified gases during subsequent phases of two-stage biofiltration–for each measurement series.

Experiment Phase	Number of Measurement Series	VOC in Raw Gases (ppb)		VOC in Purified Gases (ppb)	
		Average Value ± SD	Range	Average Value ± SD	Range
CB + MI	5	1400 ± 270	1130 ÷ 1670	125 ± 8	110 ÷ 140
	6	1170 ± 448	670 ÷ 1535	12 ± 4	10 ÷ 20
	7	480 ± 130	350 ÷ 610	24 ± 5	20 ÷ 30
	mean ± SD	1017 ± 479	480 ÷ 1400	54 ± 62	12 ÷ 125
CB + MII	8	9400 ± 1770	7630 ÷ 11170	1220 ± 11	1210 ÷ 1240
	9	7700 ± 1200	6500 ÷ 8900	283 ± 9	270 ÷ 300
	10	4700 ± 600	4100 ÷ 5300	23 ± 5	20 ÷ 30
	mean ± SD	7267 ± 2380	4700 ÷ 9400	509 ± 630	23 ÷ 1220
CBC + MI	15	990 ± 170	820 ÷ 1160	10 ± 0	-
	16	3300 ± 1180	2120 ÷ 4480	147 ± 8	140 ÷ 160
	17	7680 ± 1690	5990 ÷ 9370	127 ± 8	110 ÷ 140
	mean ± SD	3990 ± 3398	990 ÷ 7680	95 ± 74	10 ÷ 147
CBC + MII	18	1660 ± 170	1490 ÷ 1830	50 ± 5	40 ÷ 60
	19	1680 ± 160	1520 ÷ 1840	20 ± 7	10 ÷ 30
	20	1580 ± 710	860 ÷ 2280	80 ± 7	70 ÷ 90
	mean ± SD	1640 ± 53	1580 ÷ 1680	50 ± 30	20 ÷ 80

## Abbreviations

BAT	Best Available Techniques
CB	mix of stumpwood chips and bark
CBC	mix of stumpwood chips, bark and compost
ePTFE	Expanded polytetrafluoroethylene
PID	photoionization gas detector
SD	standard deviation
VOCs	volatile organic compounds
